# Crystal structure of 8-hy­droxy­quinoline: a new monoclinic polymorph

**DOI:** 10.1107/S1600536814016110

**Published:** 2014-08-01

**Authors:** Raúl Castañeda, Sofia A. Antal, Sergiu Draguta, Tatiana V. Timofeeva, Victor N. Khrustalev

**Affiliations:** a Department of Chemistry & Biology, New Mexico Highlands University, 803 University Avenue, Las Vegas, NM 87701, USA; b X-Ray Structural Centre, A.N. Nesmeyanov Institute of Organoelement Compounds, Russian Academy of Sciences, 28 Vavilov Street, B-334, Moscow 119991, Russian Federation

**Keywords:** 8-hy­droxy­quinoline, hydrogen bonds, polymorphism, crystal structure

## Abstract

In an attempt to grow 8-hy­droxy­quinoline–acetamino­phen co-crystals from equimolar amounts of conformers in a chloro­form–ethanol solvent mixture at room temperature, the title compound, C_9_H_7_NO, was obtained. The mol­ecule is planar, with the hy­droxy H atom forming an intra­molecular O—H⋯N hydrogen bond. In the crystal, mol­ecules form centrosymmetric dimers *via* two O—H⋯N hydrogen bonds. Thus, the hy­droxy H atoms are involved in bifurcated O—H⋯N hydrogen bonds, leading to the formation of a central planar four-membered N_2_H_2_ ring. The dimers are bound by inter­molecular π–π stacking [the shortest C⋯C distance is 3.2997 (17) Å] and C—H⋯π inter­actions into a three-dimensional framework. The crystal grown represents a new monoclinic polymorph in the space group *P*2_1_/*n*. The mol­ecular structure of the present monoclinic polymorph is very similar to that of the ortho­rhom­bic polymorph (space group *Fdd*2) studied previously [Roychowdhury *et al.* (1978 ▶). *Acta Cryst.* B**34**, 1047–1048; Banerjee & Saha (1986 ▶). *Acta Cryst.* C**42**, 1408–1411]. The structures of the two polymorphs are distinguished by the different geometries of the hydrogen-bonded dimers, which in the crystal of the ortho­rhom­bic polymorph possess twofold axis symmetry, with the central N_2_H_2_ ring adopting a butterfly conformation.

## Related literature   

For general background on cocrystallization of organic compounds, see: Bernstein (2002 ▶); Desiraju (2003 ▶); Dunitz (2003 ▶); Timofeeva *et al.* (2003 ▶); Aakeröy *et al.* (2009 ▶); Lemmerer *et al.* (2011 ▶). For cocrystallization of 8-hy­droxy­quinoline with different mol­ecules, see: Prout & Wheeler (1967 ▶); Castellano & Prout (1971 ▶); Liu & Meng (2006 ▶); Westcott *et al.* (2009 ▶). For crystal structure of the ortho­rhom­bic polymorph of 8-hy­droxy­quinoline, see: Roy­chowdhury *et al.* (1978 ▶); Banerjee & Saha (1986 ▶).
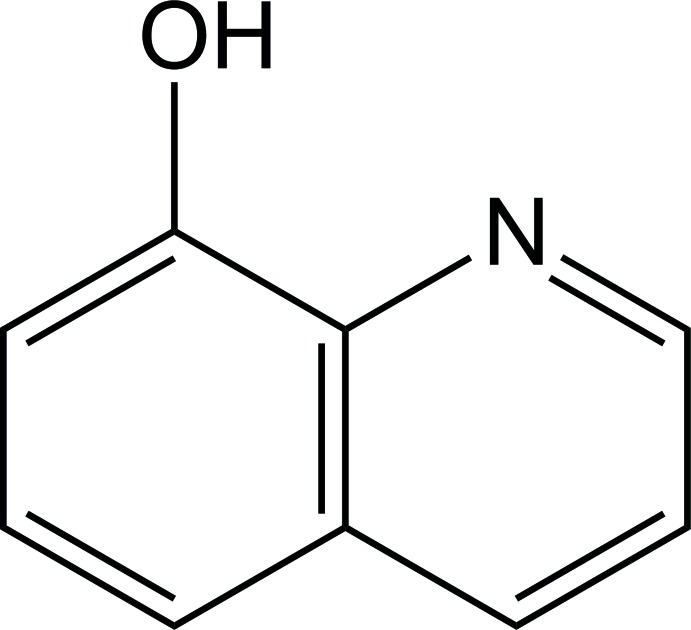


## Experimental   

### Crystal data  

C_9_H_7_NO
*M* *_r_* = 145.16
Monoclinic, 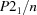

*a* = 6.620 (3) Å
*b* = 9.243 (4) Å
*c* = 11.070 (4) Å
β = 90.718 (6)°
*V* = 677.3 (5) Å^3^
*Z* = 4
Mo *K*α radiation
μ = 0.09 mm^−1^
*T* = 100 K
0.30 × 0.25 × 0.20 mm

### Data collection  

Bruker APEXII CCD diffractometer
Absorption correction: multi-scan (*SADABS*; Bruker, 2003 ▶) *T*_min_ = 0.972, *T*_max_ = 0.981
7049 measured reflections
1795 independent reflections
1494 reflections with *I* > 2σ(*I*)
*R*_int_ = 0.023

### Refinement  

*R*[*F* ^2^ > 2σ(*F* ^2^)] = 0.039
*wR*(*F* ^2^) = 0.109
*S* = 1.08
1795 reflections
103 parameters
H atoms treated by a mixture of independent and constrained refinement
Δρ_max_ = 0.39 e Å^−3^
Δρ_min_ = −0.20 e Å^−3^

### 

Data collection: *APEX2* (Bruker, 2005 ▶); cell refinement: *SAINT* (Bruker, 2001 ▶); data reduction: *SAINT*; program(s) used to solve structure: *SHELXTL* (Sheldrick, 2008 ▶); program(s) used to refine structure: *SHELXTL*; molecular graphics: *SHELXTL*; software used to prepare material for publication: *SHELXTL*.

## Supplementary Material

Crystal structure: contains datablock(s) global, I. DOI: 10.1107/S1600536814016110/rk2430sup1.cif

Structure factors: contains datablock(s) I. DOI: 10.1107/S1600536814016110/rk2430Isup2.hkl

Supporting information file. DOI: 10.1107/S1600536814016110/rk2430Isup3.cml

**Fig. 1.**
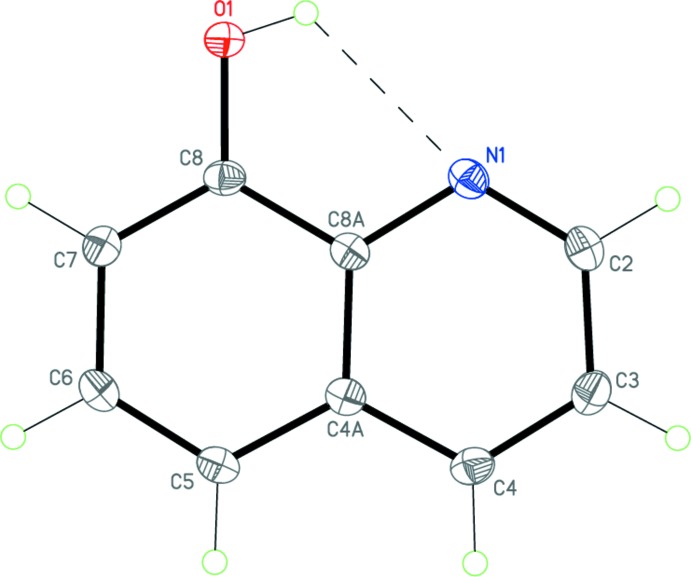
Molecular structure of I. Displacement ellipsoids are presented at the 50% probability level. H atoms are depicted as small spheres of arbitrary radius. The intramolecular O—H···N hydrogen bond is drawn by dashed line.

I . DOI: 10.1107/S1600536814016110/rk2430fig1.tif
Mol­ecular structure of **I**. Displacement ellipsoids are presented at the 50% probability level. H atoms are depicted as small spheres of arbitrary radius. The intra­molecular O—H⋯N hydrogen bond is drawn by dashed line.

**Fig. 2.**
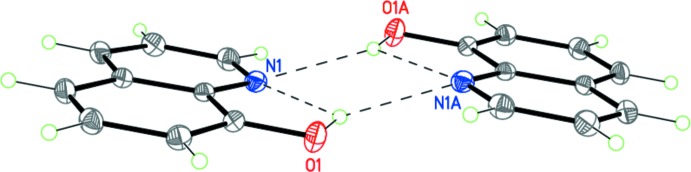
The centrosymmetric H–bonded dimers in the monoclinic polymorph of I. The hydrogen bonds are drawn by dashed lines.

I . DOI: 10.1107/S1600536814016110/rk2430fig2.tif
The centrosymmetric H–bonded dimers in the monoclinic polymorph of **I**. The hydrogen bonds are drawn by dashed lines.

**Fig. 3.**
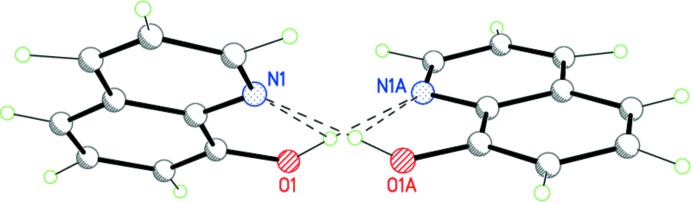
The H–bonded dimers in the orthorhombic polymorph of I, in which the molecules are related by the twofold axis. The hydrogen bonds are drawn by dashed lines.

I . DOI: 10.1107/S1600536814016110/rk2430fig3.tif
The H–bonded dimers in the ortho­rhom­bic polymorph of **I**, in which the mol­ecules are related by the twofold axis. The hydrogen bonds are drawn by dashed lines.

**Fig. 4.**
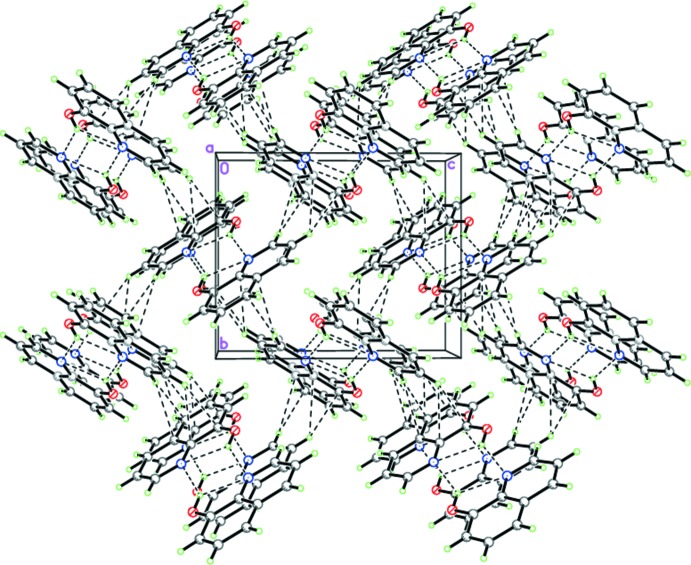
A portion of crystal packing of the H–bonded dimers in the monoclinic polymorph of I. The hydrogen bonds are drawn by dashed lines.

I . DOI: 10.1107/S1600536814016110/rk2430fig4.tif
A portion of crystal packing of the H–bonded dimers in the monoclinic polymorph of **I**. The hydrogen bonds are drawn by dashed lines.

CCDC reference: 1013310

Additional supporting information:  crystallographic information; 3D view; checkCIF report

## Figures and Tables

**Table 1:** Hydrogen-bond geometry (Å, °)

*D*—H⋯*A*	*D*—H	H⋯*A*	*D*⋯*A*	*D*—H⋯*A*
O1—H1⋯N1	0.865 (17)	2.310 (15)	2.7596 (15)	112.5 (12)
O1—H1⋯N1^i^	0.865 (17)	2.228 (17)	2.9072 (14)	135.3 (13)

Symmetry code: (i) 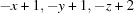.

## supplementary crystallographic information

### S1. Comment

Cocrystallization represents a form of supramolecular synthesis where molecules are linked by non–valent intermolecular interactions without making or breaking covalent bonds (Aakeröy *et al.*, 2009; Lemmerer *et al.*, 2011). Cocrystals are distinctly different from solid solutions or mixed crystals, and can be considered as molecular complexes (Desiraju, 2003; Dunitz, 2003). The ability of organic compounds to form cocrystals is dependent on a range of variables, including the types of co–formers, co–former ratios, solvents, temperature, pressure, crystallization technique *etc*. A systematic exploration of a combination of relevant variables increases the chance of discovering cocrystals with favourable properties.

In this work we attempted to prepare cocrystals of 8-hydroxyquinoline with acetaminophen by cocrystallization from chloroform–ethanol solvent mixture at room temperature. The structures of several cocrystals with 8-hydroxyquinoline have been already reported (Prout & Wheeler, 1967; Castellano & Prout, 1971; Liu & Meng, 2006; Westcott *et al.*, 2009). Unexpectedly, a new polymorph of 8-hydroxyquinoline, C_9_H_7_NO (I), was isolated, and its crystal structure was studied by X–ray diffraction analysis. However, no polymorphs of 8-hydroxyquinoline were found in Cambridge strustural database. The result presented here can be considered as a new example of so called "induced polymorphism" (Bernstein, 2002; Timofeeva *et al.*, 2003).

The molecule of I is planar, with the hydroxyl–H atom forming the intramolecular O—H···N hydrogen bond (Figure 1, Table 1). The crystal grown represents the new monoclinic polymorph in space group *P*2_1_/*n*. The molecular structure of the monoclinic polymorph of I is very close to that of the orthorhombic polymorph in space group *F*dd2 studied previously (Roychowdhury *et al.*, 1978; Banerjee & Saha, 1986). The structures of the two polymorphs are distinct by the different geometries of supramolecular synthons. In the crystals of the both polymorphs, molecules form dimers by the two intermolecular O—H···N hydrogen bonds. Thus, the hydroxyl–H atoms are involved in the bifurcated O—H···N hydrogen bonds leading to the formation of the central four–membered N_2_H_2_–ring (Table 1 for I). However, the dimers in the crystal of the monoclinic polymorph are centrosymmetrical (*C*_i_, the molecules within the dimer are parallel to each other, the central N_2_H_2_–ring is planar) (Figure 2), while those in the crystal of the orthorhombic polymorph possess the twofold axis symmetry (*C*_2,_ the molecules within the dimer are twisted by 52.4° (av.) relative to each other, the central N_2_H_2_–ring adopts a *butterfly* conformation) (Figure 3).

Further, the dimers are bound by the intermolecular π–π stacking (the interplane distance between the mean planes of closest parallel molecules in I is 3.3155 (17) Å) and C—H···π (H2···C4A^i^ 2.86 Å, H2···C5^i^ 2.87 Å; H3···C8^i^ 2.78 Å, H3···C8A^i^ 3.08 Å) (in the case of the monoclinic polymorph, Figure 4) or C—H···O (in the case of the orthorhombic polymorph) hydrogen bonding interactions into three–dimensional framework. Symmetry code: (i) 1/2-*x*, 1/2+*y*, 3/2-*z*.

### S2. Experimental

8-Hydroxiquinoline and acetaminophen were purchased from Matheson Coleman & Bell and Aldrich, respectively, and used without any further purification. 8-Hydroxyquinoline (4 mg, 27.5 mmol) and acetaminophen (4.16 mg, 27.5 mmol) were dissolved in a 1:1 chloroform–ethanol solvent mixture (3 mL). The single crystals of I were obtained by slow evaporation of the solvents at room temperature.

### S3. Refinement

The hydrogen atom of the hydroxy group was localized in the difference–Fourier maps and refined isotropically with fixed displacement parameters (*U*_iso_(H) = 1.5*U*_eq_(O)). The other hydrogen atoms were placed in calculated positions with C—H = 0.95 Å and refined within the riding model with fixed isotropic displacement parameters *U*_iso_(H) = 1.2*U*_eq_(C).

### Crystal data

**Table:**

C_9_H_7_NO	*F*(000) = 304
*M**_r_* = 145.16	*D*_x_ = 1.423 Mg m^−^^3^
Monoclinic, *P*2_1_/*n*	Mo *K*α radiation, λ = 0.71073 Å
Hall symbol: -P 2yn	Cell parameters from 2841 reflections
*a* = 6.620 (3) Å	θ = 4.2–34.9°
*b* = 9.243 (4) Å	µ = 0.09 mm^−^^1^
*c* = 11.070 (4) Å	*T* = 100 K
β = 90.718 (6)°	Prism, colourless
*V* = 677.3 (5) Å^3^	0.30 × 0.25 × 0.20 mm
*Z* = 4	

### Data collection

**Table:**

Bruker APEXII CCD diffractometer	1795 independent reflections
Radiation source: fine–focus sealed tube	1494 reflections with *I* > 2σ(*I*)
Graphite monochromator	*R*_int_ = 0.023
φ and ω scans	θ_max_ = 29.0°, θ_min_ = 4.2°
Absorption correction: multi-scan (*SADABS*; Bruker, 2003)	*h* = −9→9
*T*_min_ = 0.972, *T*_max_ = 0.981	*k* = −12→12
7049 measured reflections	*l* = −15→15

### Refinement

**Table:**

Refinement on *F*^2^	Primary atom site location: structure-invariant direct methods
Least-squares matrix: full	Secondary atom site location: difference Fourier map
*R*[*F*^2^ > 2σ(*F*^2^)] = 0.039	Hydrogen site location: difference Fourier map
*wR*(*F*^2^) = 0.109	H atoms treated by a mixture of independent and constrained refinement
*S* = 1.08	*w* = 1/[σ^2^(*F*_o_^2^) + (0.0558*P*)^2^ + 0.1943*P*] where *P* = (*F*_o_^2^ + 2*F*_c_^2^)/3
1795 reflections	(Δ/σ)_max_ = 0.001
103 parameters	Δρ_max_ = 0.39 e Å^−^^3^
0 restraints	Δρ_min_ = −0.20 e Å^−^^3^

### Special details

**Table:**

Geometry. All s.u.'s (except the s.u. in the dihedral angle between two l.s. planes) are estimated using the full covariance matrix. The cell s.u.'s are taken into account individually in the estimation of s.u.'s in distances, angles and torsion angles; correlations between s.u.'s in cell parameters are only used when they are defined by crystal symmetry. An approximate (isotropic) treatment of cell s.u.'s is used for estimating s.u.'s involving l.s. planes.
Refinement. Refinement of *F*^2^ against ALL reflections. The weighted *R*–factor w*R* and goodness of fit *S* are based on *F*^2^, conventional *R*–factors *R* are based on *F*, with *F* set to zero for negative *F*^2^. The threshold expression of *F*^2^ > 2σ(*F*^2^) is used only for calculating *R*–factors(gt) etc. and is not relevant to the choice of reflections for refinement. *R*–factors based on *F*^2^ are statistically about twice as large as those based on *F*, and *R*–factors based on ALL data will be even larger.

### Fractional atomic coordinates and isotropic or equivalent isotropic displacement parameters (Å^2^)

**Table:**

	*x*	*y*	*z*	*U*_iso_*/*U*_eq_	
O1	0.34421 (12)	0.32417 (9)	1.07956 (7)	0.0200 (2)	
H1	0.423 (2)	0.3920 (19)	1.0538 (14)	0.030*	
N1	0.32084 (13)	0.48141 (10)	0.86794 (8)	0.0156 (2)	
C2	0.30597 (17)	0.55312 (12)	0.76494 (10)	0.0176 (2)	
H2	0.4192	0.6081	0.7398	0.021*	
C3	0.13175 (17)	0.55296 (12)	0.68997 (10)	0.0180 (2)	
H3	0.1291	0.6066	0.6168	0.022*	
C4	−0.03295 (17)	0.47463 (11)	0.72420 (10)	0.0166 (2)	
H4	−0.1519	0.4741	0.6753	0.020*	
C4A	−0.02507 (15)	0.39431 (11)	0.83296 (9)	0.0139 (2)	
C5	−0.18735 (16)	0.30848 (11)	0.87370 (10)	0.0161 (2)	
H5	−0.3103	0.3046	0.8286	0.019*	
C6	−0.16632 (16)	0.23086 (12)	0.97870 (10)	0.0168 (2)	
H6	−0.2752	0.1726	1.0054	0.020*	
C7	0.01393 (16)	0.23605 (12)	1.04765 (9)	0.0159 (2)	
H7	0.0260	0.1805	1.1196	0.019*	
C8	0.17237 (16)	0.32095 (11)	1.01141 (9)	0.0146 (2)	
C8A	0.15734 (15)	0.40160 (11)	0.90195 (9)	0.0131 (2)	

### Atomic displacement parameters (Å^2^)

**Table:**

	*U*^11^	*U*^22^	*U*^33^	*U*^12^	*U*^13^	*U*^23^
O1	0.0147 (4)	0.0215 (4)	0.0238 (4)	−0.0034 (3)	−0.0053 (3)	0.0075 (3)
N1	0.0139 (4)	0.0140 (4)	0.0190 (5)	0.0000 (3)	0.0010 (3)	−0.0005 (3)
C2	0.0170 (5)	0.0162 (5)	0.0198 (5)	−0.0027 (4)	0.0025 (4)	0.0002 (4)
C3	0.0230 (6)	0.0165 (5)	0.0147 (5)	−0.0010 (4)	−0.0002 (4)	0.0016 (4)
C4	0.0180 (5)	0.0160 (5)	0.0158 (5)	0.0002 (4)	−0.0032 (4)	−0.0011 (4)
C4A	0.0139 (5)	0.0126 (5)	0.0152 (5)	0.0005 (4)	0.0002 (4)	−0.0021 (4)
C5	0.0134 (5)	0.0173 (5)	0.0175 (5)	−0.0016 (4)	−0.0011 (4)	−0.0019 (4)
C6	0.0147 (5)	0.0168 (5)	0.0190 (5)	−0.0031 (4)	0.0020 (4)	−0.0013 (4)
C7	0.0168 (5)	0.0156 (5)	0.0154 (5)	0.0004 (4)	0.0002 (4)	0.0013 (4)
C8	0.0133 (5)	0.0138 (5)	0.0166 (5)	0.0017 (4)	−0.0017 (4)	−0.0015 (4)
C8A	0.0122 (5)	0.0115 (4)	0.0157 (5)	0.0012 (3)	0.0008 (4)	−0.0021 (4)

### Geometric parameters (Å, º)

**Table:**

O1—C8	1.3575 (13)	C4A—C5	1.4139 (15)
O1—H1	0.865 (17)	C4A—C8A	1.4224 (14)
N1—C2	1.3214 (14)	C5—C6	1.3716 (16)
N1—C8A	1.3667 (14)	C5—H5	0.9500
C2—C3	1.4125 (16)	C6—C7	1.4093 (15)
C2—H2	0.9500	C6—H6	0.9500
C3—C4	1.3664 (16)	C7—C8	1.3739 (15)
C3—H3	0.9500	C7—H7	0.9500
C4—C4A	1.4149 (15)	C8—C8A	1.4250 (15)
C4—H4	0.9500		
			
C8—O1—H1	109.6 (10)	C6—C5—H5	120.2
C2—N1—C8A	117.24 (9)	C4A—C5—H5	120.2
N1—C2—C3	123.92 (10)	C5—C6—C7	121.16 (10)
N1—C2—H2	118.0	C5—C6—H6	119.4
C3—C2—H2	118.0	C7—C6—H6	119.4
C4—C3—C2	119.09 (10)	C8—C7—C6	120.38 (10)
C4—C3—H3	120.5	C8—C7—H7	119.8
C2—C3—H3	120.5	C6—C7—H7	119.8
C3—C4—C4A	119.54 (10)	O1—C8—C7	119.19 (10)
C3—C4—H4	120.2	O1—C8—C8A	120.68 (9)
C4A—C4—H4	120.2	C7—C8—C8A	120.11 (9)
C5—C4A—C4	123.08 (10)	N1—C8A—C4A	123.20 (9)
C5—C4A—C8A	119.91 (10)	N1—C8A—C8	118.01 (9)
C4—C4A—C8A	117.01 (9)	C4A—C8A—C8	118.79 (9)
C6—C5—C4A	119.63 (10)		
			
C8A—N1—C2—C3	0.76 (16)	C2—N1—C8A—C4A	−0.76 (15)
N1—C2—C3—C4	−0.05 (17)	C2—N1—C8A—C8	178.60 (9)
C2—C3—C4—C4A	−0.66 (16)	C5—C4A—C8A—N1	179.57 (9)
C3—C4—C4A—C5	−178.83 (10)	C4—C4A—C8A—N1	0.07 (15)
C3—C4—C4A—C8A	0.64 (15)	C5—C4A—C8A—C8	0.21 (14)
C4—C4A—C5—C6	178.39 (10)	C4—C4A—C8A—C8	−179.28 (9)
C8A—C4A—C5—C6	−1.07 (15)	O1—C8—C8A—N1	0.19 (15)
C4A—C5—C6—C7	0.60 (16)	C7—C8—C8A—N1	−178.25 (9)
C5—C6—C7—C8	0.77 (16)	O1—C8—C8A—C4A	179.58 (9)
C6—C7—C8—O1	179.90 (9)	C7—C8—C8A—C4A	1.14 (15)
C6—C7—C8—C8A	−1.63 (16)		

### Hydrogen-bond geometry (Å, º)

**Table:**

*D*—H···*A*	*D*—H	H···*A*	*D*···*A*	*D*—H···*A*
O1—H1···N1	0.865 (17)	2.310 (15)	2.7596 (15)	112.5 (12)
O1—H1···N1^i^	0.865 (17)	2.228 (17)	2.9072 (14)	135.3 (13)

Symmetry code: (i) −*x*+1, −*y*+1, −*z*+2.
