# Supplementary figures and images for: Crystal structure of 8-hy­droxy­quinoline: a new monoclinic polymorph
Source: Acta Crystallogr Sect E Struct Rep Online. 2014 Aug 1;70(Pt 9):o924–5. doi: 10.1107/S1600536814016110 (PMC4186174; doi:10.1107/S1600536814016110)

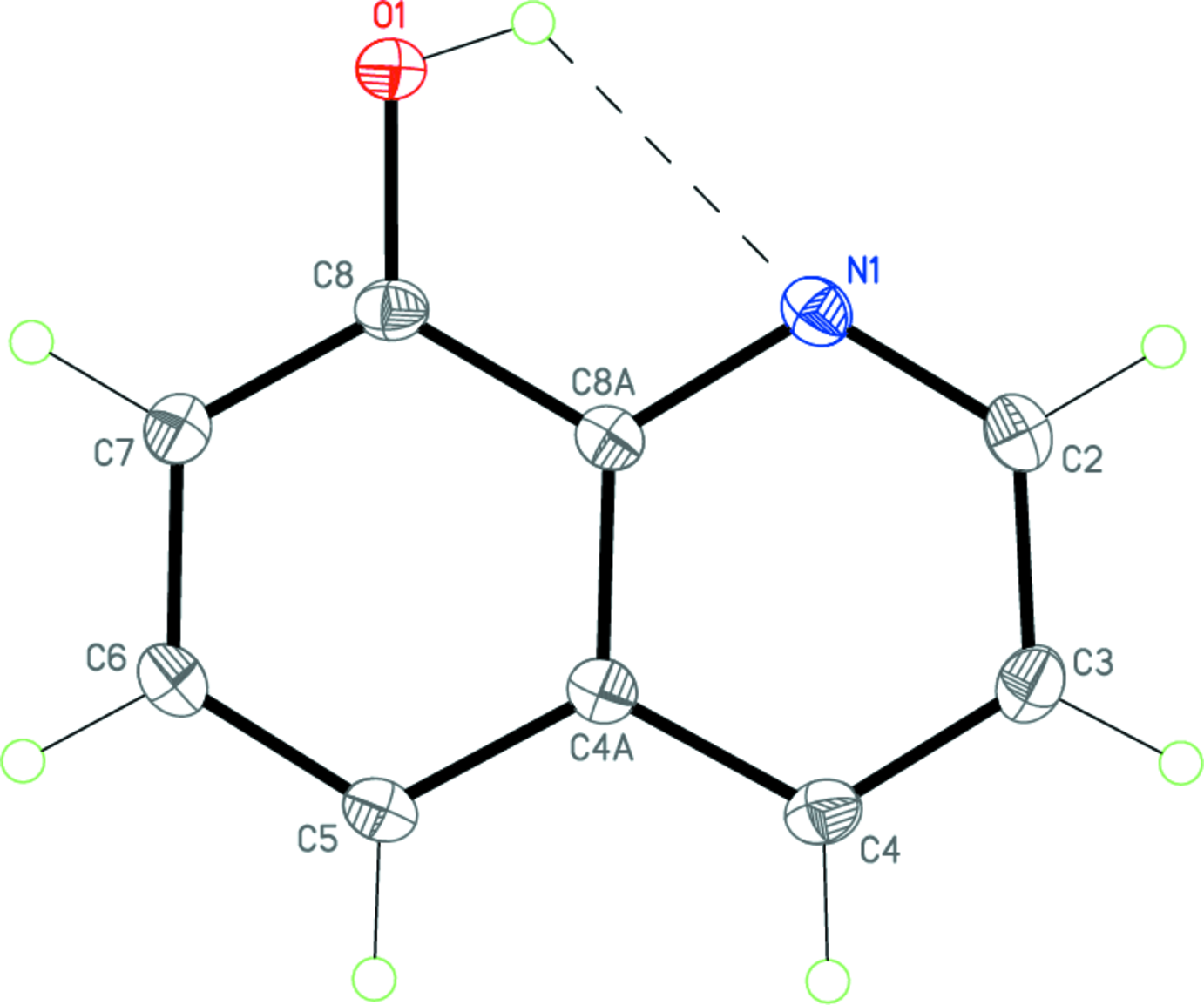

Supplement: Supplementary file 4 [file e-70-0o924-fig1.tif]

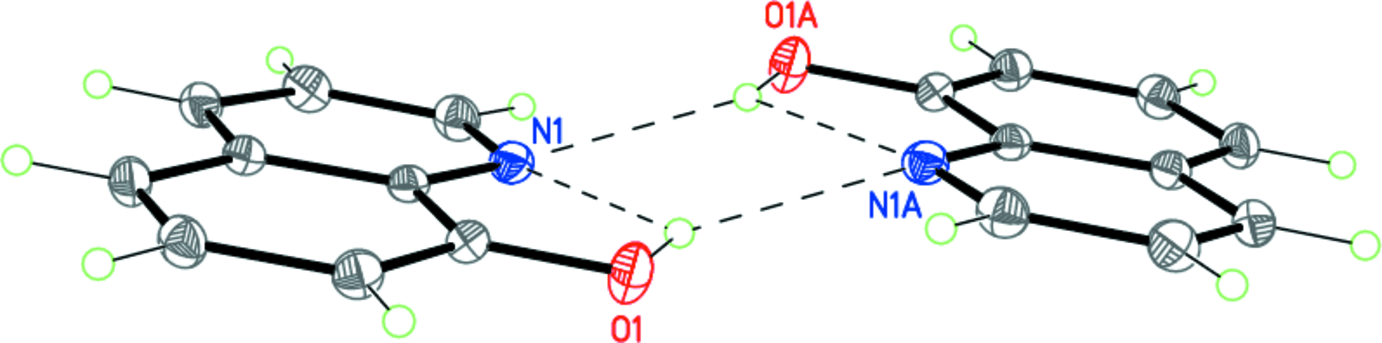

Supplement: Supplementary file 5 [file e-70-0o924-fig2.tif]

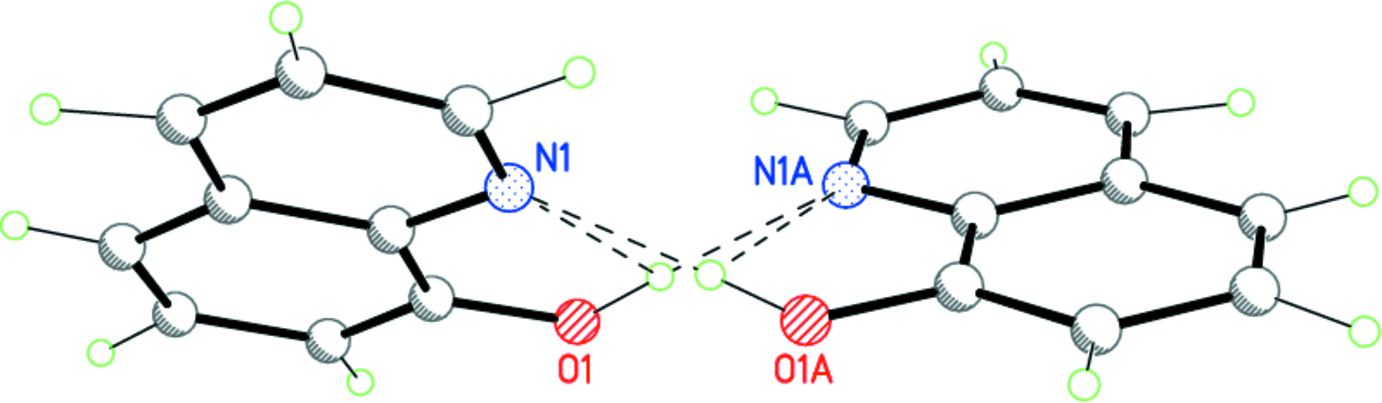

Supplement: Supplementary file 6 [file e-70-0o924-fig3.tif]

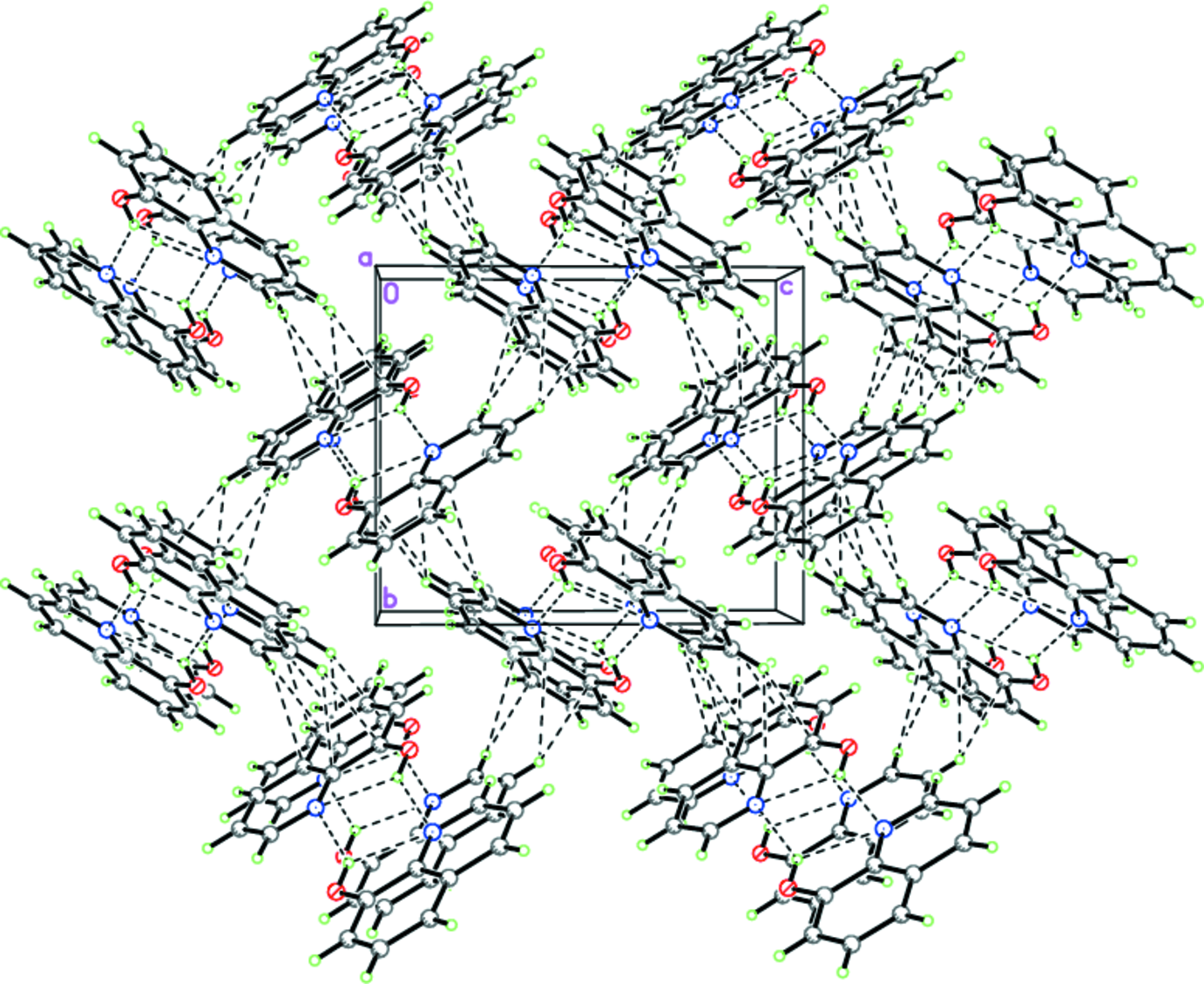

Supplement: Supplementary file 7 [file e-70-0o924-fig4.tif]
